# Insights into 4E-BP1 and p53 mediated regulation of accelerated cell senescence

**DOI:** 10.18632/oncotarget.221

**Published:** 2011-02-22

**Authors:** Suzan K. Chao, Susan Band Horwitz, Hayley M. McDaid

**Affiliations:** ^1^ Department of Molecular Pharmacology, Albert Einstein College of Medicine, Bronx, NY 10461; ^2^ Department of Medicine (Oncology) Albert Einstein College of Medicine, Bronx, NY 10461

**Keywords:** Senescence, 4E-BP1, mTORC1, p53, discodermolide

## Abstract

Senescence is a valid tumor suppressive mechanism in cancer. Accelerated cell senescence describes the growth arrested state of cells that have been treated with anti-tumor drugs, such as doxorubicin that induce a DNA damage response. Discodermolide, a microtubule-stabilizing agent, is a potent inducer of accelerated cell senescence. Resistance to discodermolide is mediated via resistance to accelerated cell senescence, and is associated with reduced expression of the mTORC1 substrate, 4E-BP1 and increased expression of p53 [1]. Although the association of p53 with senescence induction is well-characterized, senescence reversion in the presence of high expression of p53 has not been well-documented. Furthermore, studies addressing the role of mTOR signaling in regulating senescence have been limited and recent data implicate a novel, senescence-associated role for 4E-BP1 in crosstalk with the transcription factor p53. This research perspective will address these somewhat contradictory findings and summarize recent research regarding senescence and mTORC1 signaling.

## SENESCENCE

**Figure 1:**
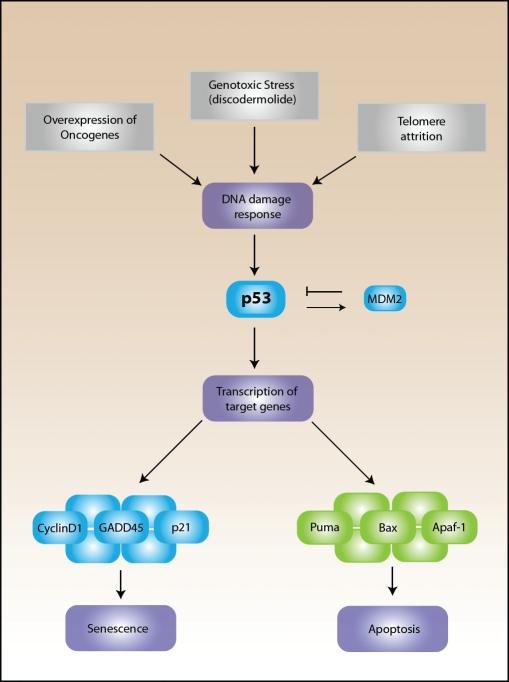
The p53 senescence pathway Several triggers such as overexpression of oncogenes, telomere dysfunction or attrition, and genotoxic stress, which includes discodermolide treatment, promote the increased activity of p53. P53 activity and stability is negatively regulated by MDM2 (Murine double minute 2) under normal conditions. Under stress conditions, the DNA damage response is activated and p53 rapidly accumulates to increase the transcription of target genes that will promote either apoptosis or senescence depending on the severity of the damage response.

Cellular senescence was first described in a study examining the proliferative potential of diploid fibroblasts that had been isolated from human fetal tissue. In this study Hayflick and Moorehead described the restricted life span of cells in culture [2]. Later, Hayflick hypothesized that the limited proliferative capacity of primary cells in culture could be the result of aging or senescence [3]. The phenomenon was later coined the “Hayflick limit”, to describe cells that had reached their maximum proliferative capacity and underwent replicative senescence. It is now known that cellular senescence is a growth arrest program that can be triggered by many stresses including telomere shortening (replicative senescence), overexpression of oncogenes such as Ras (oncogene-induced senescence), or drug-induced DNA damage (accelerated senescence) (Figure 1). However, the cellular program governing this growth arrest program is considered to be similar, regardless of the senescence trigger. This program is reported to include activation of the DNA damage response and increased p53 stability, which leads to transcription of pro-senescent genes such as p21 (Figure 1). This increased stability is the result of abrogation of the MDM2-p53 interaction. Murine double minute 2, or MDM2, is an E3 ubiquitin ligase and the major negative regulator of p53 [4]. In normal cells, MDM2 is a transcriptional target of p53 creating a negative feedback loop that maintains p53 at low levels, but during stress, p53 escapes interaction with MDM2 and accumulates in the nucleus to initiate transcription of target genes capable of inducing senescence.

## SENESCENCE AS A THERAPY FOR CANCER TREATMENT

“Accelerated cell senescence”, “premature senescence”, and “senescence-like growth arrest” are interchangeable terms that refer to the proliferative arrest observed in tumor cells when treated with an anticancer agent. It has long been appreciated that tumor cells have many different responses to chemotherapy and radiation, the best characterized of which is apoptosis that results in the clearance of affected tumor cells. This is equated clinically with either a partial or complete response to therapy, mediated by regression of tumor cells. However, many tumors are resistant to apoptosis and senescence induction is considered a viable option since senescent cells *in vivo*, are cleared by macrophages [5], thus tumor regressions may also be achieved. The caveat to this is the fact that in some situations, clearance of senescent cells does not occur, for example RAF-mutant senescent cells that comprise benign nevi. Efforts are underway to understand the various cellular contexts under which senescent cells persist or become cleared by the immune system.

Furthermore, drug-induced proliferative arrest and quiescence are clinically relevant responses that manifest as stable disease in cancer patients. Unlike senescence, quiescence is an easily reversible process and cancer cells can resume proliferation when treatment ceases, upon growth factor stimulation, or due to epigenetic mechanisms that mediate resistance to therapy [6, 7]. Senescence is considered a potent tumor suppressive mechanism *in vivo* and thus, is regarded as a negative regulator of oncogenic transformation [5, 8-12]. Thus, the induction of senescence as a treatment modality for cancer is considered a viable approach for the clinical management of malignancy, with the understanding that proliferative arrest may be the predominant mechanism. Senescence-inducing drugs may also be utilized in combination with other therapies to potentiate either apoptosis or growth arrest in tumor cells [13].

## TUBULIN-STABILIZING DRUGS AS INDUCERS OF SENESCENCE

**Figure 2:**
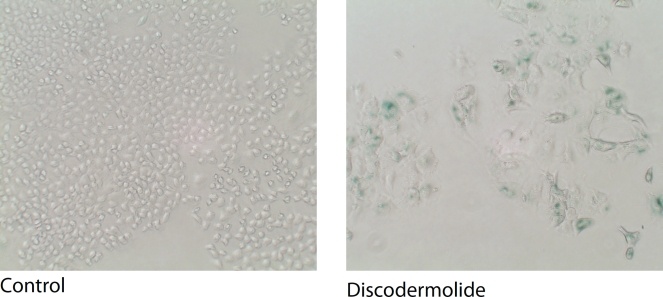
Discodermolide-induced accelerated cell senescence Senescence-associated β-galactosidase activity in A549 lung carcinoma cells treated with control (DMSO) or, an IC_50_ concentration of discodermolide for 6 days.

DNA damaging agents such as doxorubicin, cisplatin, and ionizing radiation have been well characterized as inducers of accelerated cell senescence [14]. In addition to these agents, we and others have demonstrated that microtubule-stabilizing agents such as discodermolide, and to a much lesser extent Taxol, can induce accelerated cell senescence [15, 16] (Figure 2).

Discodermolide, which was isolated from the Caribbean sea sponge *discodermia dissoluta*, is a microtubule-stabilizing agent that was originally characterized as an immunosuppressant [17-20]. Discodermolide is more water soluble than Taxol, interacts synergistically with Taxol to suppress the growth of numerous cancer cell lines [21, 22], does not exhibit cross-resistance to Taxol-resistant cells, and importantly as noted above, is a potent inducer of accelerated senescence [15]. Lastly, discodermolide resistant cells do not exhibit classical mechanisms of resistance observed in cells that have lost sensitivity to Taxol, rendering discodermolide and its analogs promising candidates for future clinical development.

Ixabepilone is an analog of epothilone B, a microtubule stabilizing agent originally isolated from the gram-negative bacteria, *Sorangium cellulosum*. It is FDA-approved for the treatment of metastatic breast cancer in taxane-refractory patients. At present, the senescent-inducing properties of epothilones are poorly characterized.

## MARKERS OF SENESCENCE

Accelerated cell senescence shares many of the well-characterized markers of replicative and oncogene-induced senescence [23, 24]. These include, a large flat cellular morphology; expression of a Senescence-Associated β-galactosidase activity (SA-β-gal) that distinguishes them from quiescent cells; formation of intracellular vacuoles; resistance to mitogenic stimulation and formation of punctuate, highly condensed facultative heterochromatin called Senescence-Associated-Heterochromatic Foci (SAHF) [25, 26]. Proteins that have increased expression during senescence that have been used as markers include cyclin D1, γH2AX, IL-8, IL-6 and IGFBPs, however upregulation of these is not universally observed [25, 27]. Senescent cells are metabolically active and translate a plethora of secretory factors that has been termed the senescence associated secretory phenotype, or SASP. The SASP is comprised of interleukins, inflammatory cytokines, proteases, and extracellular matrix components [28]. Therefore, senescent cells, unlike quiescent cells, have comparable levels of overall protein synthesis to cycling cells except that the repertoire of translated mRNAs is dramatically altered.

One interesting molecular marker of senescence is plasminogen activator inhibitor 1 (PAI-1), a direct transcriptional target of p53 [29]. PAI-1 is an inhibitor of urokinase type plasminogen activator (uPA), a secreted protease involved in extracellular matrix remodelling. Previously thought to be only a marker of the senescent phenotype, PAI-1, has been reported to be required for p53-mediated replicative senescence induction in primary mouse diploid fibroblasts and human BJ cells [30]. While PAI-1 is strongly induced in discodermolide-induced accelerated cell senescence, knockdown of PAI-1 in human tumor cells does not prevent senescence, but rather decelerates senescence induction, suggesting that in this context, PAI-1 acts as a modulator of senescence onset, an observation that is consistent with the redundant nature of senescence (Laura Klein, unpublished data).

## THE ‘PERMANENCE’ OF SENESCENCE

Although senescence is a tumor suppressive mechanism in normal cells, recent studies suggest that senescence may promote transformation via creation of a proinflammatory microenviroment by the SASP that promotes extracellular remodeling. Therefore, the tumor suppressive potential of senescence induction as a therapeutic strategy is limited by the concern that senescent cells may not be effectively cleared [27, 28, 31, 32]. Furthermore, the clinical implementation of senescent-inducing therapies also relies somewhat on the perception that senescence is irreversible, although studies, including our own, indicate that accelerated cell senescence and oncogene-induced senescence can indeed be evaded [1, 27, 33, 34]. Importantly, senescence escape, or reversion, is not to be confused with evasion, in which cells that were not initially senescent outgrow and form the majority population.

In a given population of stably senescent cells, there is presumably, a strong selection for escape. In the case of drug-induced accelerated senescence, it has been our experience that escape is challenging in cell culture, since it took several years for us to isolate a senescence-resistant line [1]. It is reasonable to assume that *in vivo*, senescent cells may be in a more permissive environment for escape, and this may contribute to tumor progression. This logic seems particularly valid if one considers the effects of senescence-associated SASP induction on the surrounding extracellular matrix, which is poorly modeled in cell culture systems.

Finally, senescent cells have areas of highly condensed facultative heterochromatin called Senescence-Associated -Heterochromatic Foci (SAHF), which are specialized domains of transcriptionally silent, senescence-associated heterochromatic foci [35]. These repress the expression of proliferation-promoting genes and several studies have implicated epigenetic alterations as important events in senescence reversion. One gene that has been implicated in senescence reversion is the methylation enzyme S-adenosylhomocysteine hydrolase, SAHH. Inactivation of SAHH permits escape from p53 and Rb-mediated replicative senescence [36]. Therefore, S-adenosylhomocysteine hydrolase downregulation contributes to tumorigenesis, reinforcing the significance of epigenetic processes in senescence and cellular transformation.

## SENESCENCE, CANCER EVOLUTION AND DIFFERENTIATION

Cancer development is a multistep process and senescent tumor cells, if not cleared by phagocytosis, are under strong selective pressure to revert and may gradually acquire alterations that enable them to re-enter the cell cycle. As discussed previously, pro-inflammatory signaling originating from senescent tumor cells, may also promote localized transformation in neighboring cells that manifests as tumor progression *in vivo*. This may rationalize the observation that tumors that initially respond to chemotherapy treatment often become resistant to drugs. Clonal expansion of these cells is believed to contribute to the progression of drug-resistant tumors. The emergence of a drug-resistant population from a senescent precursor has been termed neosis [37, 38]. Future studies to identify factors that make this escape possible are crucial to understanding both proliferation cues in the cell and cancer progression.

Furthermore, it is well known that cancer cell lines and tumors are genetically heterogeneous and this observation also applies to the basal level of senescence in a given cancer cell population. Specifically, in some breast cancer cell lines, senescent cells have been identified by SA-β-gal positivity, suggesting that the senescence machinery is intact in some tumors. Interestingly, high numbers of senescent cells exist in estrogen receptor positive-expressing breast cancer cell lines that are models for treatment-responsive luminal A and B disease. Conversely, substantially fewer senescent cells are found in breast cancer cell lines that are basal-like, which represent patients with disease that although chemo-responsive, is at high risk for relapse [39]. These data suggest that the basal level of senescence in a tumor reflects (a) the capacity for proliferation, and (b) the differentiation status, which for breast cancer, guides the choice of treatment, and also predicts outcome to therapy.

## THE KEY PLAYERS: MTOR, 4E-BP1, AND P53

**Figure 3:**
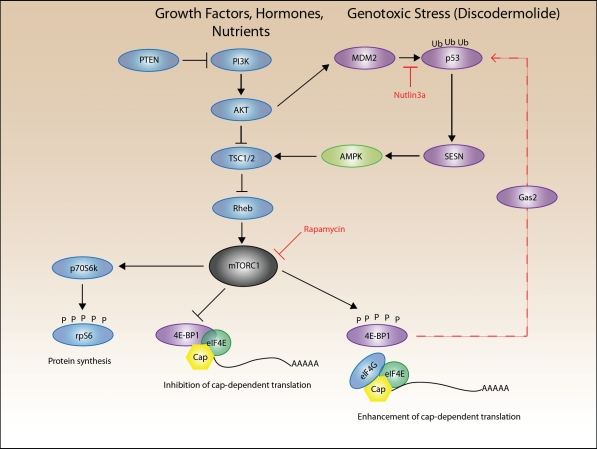
A simplified schematic of the PI3K/Akt/mTORC1 pathway Growth factors or hormones can stimulate phosphatidylinositol 3-kinase (PI3K) signaling. Additionally, PTEN is commonly mutated in cancer, causing increased Akt activity and signaling through mTORC1. Activation of PI3K activates Akt, which can phosphorylate TSC2 leading to the inactivation of the TSC1/2 inhibitor complex. Released from inhibition, Rheb can then activate mTORC1. Activated mTORC1 phosphorylates its downstream substrates p70S6k and 4E-BP1. Phosphorylation of p70S6k results in phosphorylation of rpS6. mTORC1 phosphorylation of 4E-BP1 releases 4E-BP1 from eIF4E on the 5' cap of mRNA, and enhances cap-dependent translation. 4E-BP1 inhibits translation of Gas2, which increases the stability of p53 by binding m-calpain and inhibiting its protease activity towards p53. Genotoxic stress activates p53, which induces the transcription and expression of SESNs (SESN1 and SESN2). SESN phosphorylates and forms a complex with AMPK and TSC2 that results in the phosphorylation of TSC2, eventually leading to activation of mTORC1 and its substrates 4E-BP1 and p70S6k.

The PI3K/Akt/mTOR signaling pathway is important in cancer progression and regulates metabolism, cell survival and cell growth [40] (Figure 3). The mammalian target of rapamycin (mTOR) pathway exists as two protein complexes in the cell, mTOR complex 1 and 2 (mTORC1 and mTORC2, respectively), reviewed in [41]. Although the function of these complexes is still an area of active research, mTORC1, which contains mTOR, Raptor, mLST8 and PRAS40, regulates protein synthesis and cell proliferation. Conversely, mTORC2, contains mTOR, Sin1, Rictor, mLIST8 and PROTOR, and primarily regulates movement of the actin cytoskeleton and cell spreading. Rapamycin is an immunosuppressant that inhibits mTORC1 via binding to FKBP12 and together this complex binds and inhibits mTORC1 Ser/Thr kinase activity. Previously, it was thought that mTORC2 was rapamycin insensitive; however, prolonged treatment with rapamycin decreases mTORC2 complex formation [42]. Studies investigating the role of mTOR in senescence have been limited, and focused on its known regulation of proliferation. Rapamycin has been reported to suppress senescence in a variety of cell lines [43, 44] and also has anti-aging effects that can increase life span in mice [45]. Additionally, fasting or caloric restriction is known to decrease mTORC1 activity and contribute to longevity. A recent study by Sengupta et al. demonstrates that mTORC1 activity decreases the production of ketones by the liver, which is associated with aging, and suggests that the effects of mTORC1 activity on aging can be attributed to mTORC1's role as a nutrient sensor [46].

4E-BP1 (eIF4E binding protein 1) is a downstream substrate of mTORC1 that regulates cap-dependent translation. 4E-BP1 undergoes hierarchical phosphorylation by mTORC1 leading to its activation, reviewed in [47]. Inhibition of mTORC1 by drugs such as rapamycin dephosphorylates 4E-BP1, thereby enhancing its association with the mRNA 5' cap-binding protein, eIF4E, and suppressing cap-dependent translation (Figure 3). Conversely, 4E-BP1 may be hyperphosphorylated by activated mTOR, leading to its dissociation from eIF4E and enhanced translation of a specific subset of growth promoting mRNAs [48]. eIF4E acts oncogenically, and when overexpressed induces senescence independently of other stimuli in primary cells. It is overexpressed in various malignancies [49, 50], however, the mechanism by which eIF4E induces oncogenic transformation is not well understood.

In our recent paper we describe a cell line, AD32 that is resistant to senescence [1]. This resistance is dependent upon 4E-BP1, as re-expression of 4E-BP1 reverted resistance to the senescence-inducer discodermolide. It has been previously demonstrated that p53 controls the dephosphorylation of 4E-BP1 and inhibition of translation through mTORC1-dependent effects [44, 51, 52]. Later, it was discovered that activation of p53 led to increased transcription of negative regulators of mTORC1 such as PTEN, AMPKβ, and TSC2 [53, 54]. The precise mechanism for the p53-mediated activation of AMPK was unknown until Budanov et al. demonstrated that p53 initiates the transcription of sestrins in response to genotoxic stress [55]. The sestrin family of cytoplasmic proteins consists of SESN1, SESN2, and SESN3, all of which function in antioxidant defense by regenerating peroxiredoxins. In particular, SESN1 and SESN2 are able to negatively regulate mTOR, a redox sensitive kinase. However this was independent of their redox activity. Since redox-impaired mutants were able to suppress mTORC1 as efficiently as wild type [55]. Importantly, sestrins mediate mTORC1 suppression via AMPK activation, which in turn phosphorylates TSC2, a negative regulator of mTORC1. Furthermore, AMPK can phosphorylate p53 at Ser15, a site that enhances p53 stability and activation [56, 57]. This AMPK-mediated p53 stabilization may result in a positive feedback loop, further indicating the importance of p53 and mTORC1 signaling in response to genotoxic stress [58]. In addition, 4E-BP1 has been shown to control the translation of Gas2, a protein that regulates p53 stability and senescence [59].

AD32 cells, which have escaped senescence, express high levels of stable p53 protein relative to the senescence-sensitive precursor cell line, A549. In addition, we demonstrated that with increasing discodermolide resistance, p53 protein expression increased, while 4E-BP1 expression decreased [1]. While our findings support previous studies that had established a relationship between 4E-BP1 and p53 [51, 52, 59-61], the finding that increased levels of stabilized p53 was not associated with senescence, but rather, escape from senescence, was paradoxical.

This finding led us to ponder the current paradigm that places p53 as an essential component in accelerated cell senescence. Increased p53 activity is a hallmark of cell senescence, but does p53 drive senescence induction? Can senescence happen in the absence of p53? Or, is it possible that p53 suppresses senescence? Links between p53, p21 and cellular senescence have been well established. It is certainly true in some primary mouse embryonic fibroblasts (MEFs), that replicative senescence requires p53 and cells can be immortalized by loss of p53, p19ARF, or Rb proteins [11]. However, differences in basal senescence between cell lines do exist, just as differences in the propensity for senescent cells to exhibit a SASP phenotype exist. For example, human cells seem to be more dependent upon p21 for growth arrest, whereas p21 is not essential for senescence in mouse fibroblasts [62]. Additionally, there are two known tumor suppressor pathways that regulate the senescence response, the p53 pathway and the p53-independent or p16INK4a/RB pathway. Each of these pathways integrates a variety of stress signals that determine whether a cell undergoes senescence or apoptosis. In many cancer cell lines, p53 is mutated or mislocalized and p16 is epigenetically silenced; yet these cells are still able to execute the senescence program [16].

Recent studies, including our own, demonstrate that senescence can occur in cells that have compromised p53 [15, 43, 63-65]. A recent study by Demidenko et al. indicates that p53 may act as a suppressor of senescence in certain contexts [44]. This model provides a possible explanation for the reversion of accelerated cell senescence that leads to the generation of AD32 cells, despite high expression of p53 and p21. In this study [44], cells were engineered to conditionally overexpress p21 and it was found that overexpression of p53 drove quiescence, while p21 drove senescence. Cells induced to overexpress ectopic p21 became senescent, but this could be converted to quiescence by p53 overexpression, indicating that p53-driven suppression of the senescent phenotype may override senescence driven by p21. Furthermore, rapamycin was able to suppress the senescent phenotype, and also nutlin3a, an MDM2 antagonist that stabilizes p53. Interestingly, AD32 cells are cross resistant to rapamycin but have wild-type 4E-BP1 function, capable of binding eIF4E [1]. We have discovered that 4E-BP1 expression modulates senescence, as restoration of expression made these cells susceptible to discodermolide-induced accelerated cell senescence. Partial knockdown of p53 in AD32 cells had essentially no effect on discodermolide-induced senescence or cytotoxicity, thereby indicating that 4E-BP1, in this system, may play a more significant role in the accelerated cell senescence response than p53.

## FUTURE DIRECTIONS

It is known that eIF4E acts oncogenically if overexpressed, resulting in tumor growth [49]. Presumably, its oncogenic activity lies in its ability to direct the translation of specific mRNAs that participate in advancing the malignant phenotype [66]. Identification of transcripts that are bound by eIF4E has been elusive, with most studies utilizing eIF4E overexpression screens [67, 68]. The putative mRNA targets that have been discovered include cMYC, cdk2, cyclinD1, MMP9, Mcl-1, Bcl-2, survivin, VEGF, and FGF2 [66, 69-71]. Many of the mRNAs identified play crucial roles in cell growth, metastasis, angiogenesis, and cell survival. Undoubtedly, there are vastly more that remain to be identified. The mRNAs regulated by eIF4E are considered “weak” mRNAs since they are generally poorly translated and associate with the monosome fraction in polysome gradients, likely due to their highly structured 5' UTRs. “Weak” mRNAs include proto-oncogenes and growth factors, whereas “strong” mRNAs, include housekeeping genes, reviewed in [50]. Thus far, the precise mechanism that eIF4E utilizes to target particular mRNAs is unknown. It would be of value to characterize the repertoire of transcripts bound to eIF4E under different conditions and in different tissues. It has been demonstrated that eIF4E is phosphorylated by MNK1 and MNK2 kinases and may be regulated by other kinases, depending on nutrient, or growth signals [72, 73]. As the main inhibitor of eIF4E, and a direct substrate of mTORC1, future studies should focus on transcripts inhibited by 4E-BP1.

To this end, we performed transcriptome analysis on 4E-BP1 overexpressing cell lines and identified several changes in p53 response genes and those involved in the DNA damage response, supporting a role for discodermolide as a senescence-inducer that elicits a DNA damage response. Since p53 is a pleiotropic signaling molecule that participates in a multitude of cellular processes, it is plausible that the elevated expression observed in AD32 cells simply reflects a high basal level of DNA damage that the cells acquired while senescent, which can be tolerated by the revertant AD32 population. In this model, AD32 cells may have adjusted the internal p53 ‘rheostat’ to proliferate in the presence of high p53 expression.

Others have used polysomal fractionation combined with microarray analysis to identify those transcripts that are more efficiently translated [68, 74]. While these approaches enrich for mRNAs that are associated with ribosomes, they do not directly identify transcripts bound to eIF4E and 4E-BP1, or associated proteins that may contribute to eIF4E's specificity for particular mRNAs. For instance, CPEB is a RNA-binding protein that recognizes and binds a specific sequence in the 3' UTR of mRNA in *Xenopus laevis*. The *Xenopus* 4E-BP, maskin, interacts with CPEB and together with other proteins regulates the translation of mRNA [75, 76]. A study in *Saccharomyces cerevisiae* has identified PUF proteins, mRNA binding proteins that interact with the yeast 4E-BPs, eap1 and caf20. PUF proteins in combination with 4E-BPs mediate translation of a specific subset of mRNAs [74]. Recent studies employing RNA-Binding Protein Immunoprecipitation-Microarray Profiling (RIp-Chip or ribonomic profiling) were able to identify RNP complexes that specifically associated with mRNAs that shared biological function or activity [77-80]. Future studies will be able to identify which mRNAs are inhibited by 4E-BP1 and possible cognate factors that facilitate this specificity, and changes in the population of transcripts bound during senescence.

We cannot rule out the possibility that 4E-BP1 is a multifunctional protein, that regulates senescence in an mTORC1-independent manner. Overexpression of a 4E-BP1 Thr37/46Ala nonphosphorylatable mutant in AD32 cells was able to partially revert resistance to discodermolide, indicating that the function of 4E-BP1 in senescence may be independent from mTORC1 phosphorylation. Although rapamycin universally dephosphorylates the mTORC1 substrate S6K, it does not cause 4E-BP1 dephosphorylation in every cell type [81], nor does it result in dissociation of the mTORC2 complex in every case. These observations have led to the hypothesis that clinical response to rapalogs occurs in tumors that have dephosphorylation of 4E-BP1 and S6K, and mTORC2 dissociation, although this has yet to be substantiated. Several groups, including ours have suggested regulation of 4E-BP1 by additional kinases, reviewed in [82]. In fact, we have clearly demonstrated that dual suppression of the RAS-PI3K by combined MEK and rapalog treatment is highly synergistic and that mechanistically this is mediated via potent suppression of cap-dependent translation and dephosphorylation of S6 and 4E-BP1 [83].

A less well-characterized role of 4E-BP1 is its ability to regulate the subcellular localization of eIF4E, as ~30% of 4E-BP1 is localized to the nucleus [84]. Importantly, nuclear localization is prevented in the presence of oncogenic RAS, although this mechanism is not well understood. 4E-BPs do not have nuclear localization or export motifs, so it appears that the nucleocytoplasmic trafficking of the protein may be regulated by RAS. These studies provide further evidence of mTORC-independent functions of 4E-BP1.

Phosphorylation of Ser15 of p53 abrogates the p53-MDM2 interaction, and AD32 cells have increased phosphorylation of Ser15. However, both MDM2 and p53 are regulated by various kinases. For example, MDM2 undergoes phosphorylation by AKT, a cell survival factor, at Ser166. Phosphoryation of MDM2 results in increased E3 ligase activity, targeting p53 for degradation. It is unclear how during DNA damage, p53 is able to elude ubiquitination by MDM2 so rapidly. Our cells contain increased levels of both MDM2 and p53. It has recently been discovered that microRNA 605 (mir605) regulates the p53-MDM2 interaction. Mir605 is a transcriptional target of p53 and participates in a positive feedback loop by degrading MDM2 [85]. This mechanism provides another example of the complexity of the senescence response. Adding to the complexity, mir605 when overexpressed preferentially induces apoptosis rather than senescence [85]. It is plausible that there may be other miRNAs capable of regulating senescence. With each new discovery about p53 regulation, new possibilities arise, suggesting that there is a delicate balance between tumor suppression and oncogenesis with multiple levels of regulation.
